# MYBL2-induced PITPNA-AS1 upregulates SIK2 to exert oncogenic function in triple-negative breast cancer through miR-520d-5p and DDX54

**DOI:** 10.1186/s12967-021-02956-6

**Published:** 2021-08-05

**Authors:** Bolong Liu, Pingbo Yao, Feng Xiao, Jianjin Guo, Lianghui Wu, Yong Yang

**Affiliations:** 1 Department of Andrology, The First Affiliated Hospital of University of South China, Hengyang, 421001 Hunan China; 2 Changsha Social Work College, Changsha, 421004 Hunan China; 3 Department of Oncology, The Second Hospital of Shanxi Medical University, Taiyuan, 030001 Shanxi China; 4 Department of Endocrinology and Metabolism, The Second Hospital of Shanxi Medical University, Taiyuan, 030001 Shanxi China; 5 Department of Intensive Care Unit, Affiliated Nanhua Hospital, University of South China, No. 336, Dongfeng South Road, Zhuhui District, Hengyang, 421001 Hunan China; 6 Department of General Surgery, The Second Hospital, University of South China, 30 Jiefang Road, Shigu District, Hengyang, 421001 Hunan China

**Keywords:** PITPNA-AS1, miR-520d-5p, DDX54, SIK2, TNBC

## Abstract

**Background:**

In recent years, long non-coding RNAs (lncRNAs) have attracted much attention because of its regulatory role in occurrence and progression of tumors, including triple-negative breast cancer (TNBC). LncRNA PITPNA antisense RNA 1 (PITPNA-AS1) has been explored in some cancers, whereas its function and molecular mechanism in TNBC remain unclear.

**Methods:**

PITPNA-AS1 expression in TNBC tissues and cells was determined by RT-qPCR. TNBC cell viability, proliferation, migration, invasion were assessed with CCK-8, colony formation, wound healing, transwell assays. Cell apoptosis was evaluated by flow cytometry. Expression of EMT-related markers was detected by western blot analyses. The molecular mechanism of PITPNA-AS1 was explored by RNA pull down, luciferase reporter, RIP and ChIP assays.

**Results:**

PITPNA-AS1 showed high expression levels in TNBC tissues and cells. PITPNA-AS1 knockdown suppressed TNBC cell viability, proliferation, migration, invasion in vitro and inhibited xenograft tumor growth in mice. Mechanistically, PITPNA-AS1 upregulated SIK2 expression by sponging miR-520d-5p and recruiting DDX54 protein. Results of rescue assays suggested that the inhibitive effects of silenced PITPNA-AS1 on TNBC cellular processes were partially rescued by overexpressing SIK2 or combination of miR-520d-5p inhibition and DDX54 overexpression. More importantly, we found that the upregulation of PITPNA-AS1 in TNBC cells was attributed to transcription factor MYBL2.

**Conclusion:**

PITPNA-AS1 activated by MYBL2 plays an oncogenic role in TNBC through upregulating SIK2.

**Supplementary Information:**

The online version contains supplementary material available at 10.1186/s12967-021-02956-6.

## Introduction

There is a rapid rising trend in the incidence of breast cancer (BC) with more than 1 million new-diagnosed cases each year [1]. According to molecular characteristics of HER-2, estrogen receptor (ER), ki-67, and progesterone receptor (PR), BC can be divided into diverse subtypes, such as HER-2 overexpression, Lumina A, Lumina B, ‘normal-like’ breast tumors and basal-like tumors. As defined, triple-negative breast cancer (TNBC) is a tumor that is negative for HER-2, ER and PR [2]. Currently, surgical resection and chemotherapy are the major systemic therapies for patients with TNBC due to the lack of effective biomarker [3]. Compared with patients with other BC subtypes, TNBC patients have significantly higher rates of metastasis and recurrence [4, 5]. Thus, it is essential to explore more effective biomarkers for early diagnosis and advanced treatment of TNBC.

It was commonly accepted that non-coding RNAs (ncRNAs) act as critical factors in the regulation of gene expression, thereby exerting functions on tumor or non-tumor cell phenotypes [6, 7]. Long non-coding RNAs (lncRNAs) are generally regarded as genomic transcripts exceeding 200 nucleotides in length [8]. Lacking open reading structure of indispensable length, lncRNAs are limited in protein-coding [9]. It has been highlighted by diverse researches that lncRNAs are associated with molecular mechanisms underlying cancer development and can be used as promising biomarkers and therapeutic targets for cancers [10]. Previously, many studies have emphasized the important roles of lncRNAs in regulating various cell biological behaviors including cell growth, apoptosis, migration and invasion [11, 12]. Increasing lncRNAs, such as NEAT1 [13], BORG [14] and LINC01638 [15], have been identified as oncogenes in TNBC.

LncRNAs are implicated in tumorigenesis and progression of cancers via multiple mechanisms [16]. Importantly, lncRNA can function as a competing endogenous RNA (ceRNA) to upregulate mRNA expression by sponging microRNA (miRNA). For example, lncRNA EPB41L4A-AS2 upregulates FOXL1 expression to suppress hepatocellular carcinoma progression via sponging miR-301a-5p [17]. LncRNA NORAD facilitates cervical cancer cell proliferation and invasion by targeting the miR-590-3p/SIP1 axis [18]. In addition, lncRNAs can modulate tumor initiation by binding with RNA-binding proteins (RBPs) to maintain mRNA stability. For instance, LINC00324 positively regulates FAM83B mRNA stability to promote cell proliferation in gastric cancer through binding with HuR protein [19]. LBX2-AS1 stabilizes LBX2 mRNA expression to drive gastric cancer progression via recruiting FUS protein [20]. LncRNA PITPNA antisense RNA 1 (PITPNA-AS1) has been previously reported as an oncogene in hepatocellular carcinoma by acting as a ceRNA [21]. However, its functional role and molecular mechanism in TNBC remain unclear.

In this research, we investigated the function and regulatory mechanism of PITPNA-AS1 in TNBC cellular processes. Our findings revealed that MYBL2-activated PITPNA-AS1 sponged miR-520d-5p and recruited DDX54 protein to increase SIK2 expression, thereby promoting cellular activities in TNBC. This finding implicated PITPNA-AS1 as a promising biomarker for TNBC treatment.

## Materials and methods

### Tissue samples

Total 56 pairs of TNBC tissues and their adjacent non-tumor tissues were collected from patients who were diagnosed with TNBC at the Second Hospital of Shanxi Medical University (Shanxi, China). Before the surgery, all patients have signed informed consents and none of them received any anti-cancer treatments. The specimens collected from the patients were snap-frozen in liquid nitrogen and subsequently preserved at − 80 °C. Gene expression analysis using 56 pairs of TNBC tissues and corresponding non-tumor tissues was conducted, which was approved by Institutional Ethics Committees of the Second Hospital of Shanxi Medical University.

### Cell lines

One normal breast epithelial cell, MCF10A, and four human TNBC cell lines (HCC1937, MDA-MB-468, MDA-MB-231, MDA-MB-436) were bought from ATCC (Manassas, VA). Cells were incubated in DMEM (Invitrogen, Carlsbad, CA) containing 10% fetal bovine serum (FBS) and 1% penicillin/streptomycin in a humidified air at 37 °C with 5% CO_2_.

### Cell transfection

HCC1937 and MDA-MB-468 cells were transfected with the sh-RNAs (plasmid backbone: pGPU6/Hygro) targeting PITPNA-AS1 (sh-PITPNA-AS1#1/2), DDX54 (sh-DDX54#1/2), MYBL2 (sh-MYBL2) and corresponding negative controls (sh-NC) at the final concentration of 100 nM. Stably transfected cells were selected by detection of Hygromycin B resistance. To overexpress SIK2, E2F6, PAX5, FOXP2, ELK5, MYBL2 and YY1, pcDNA3.1 vector targeting corresponding genes and empty pcDNA3.1 vector (control) were transfected into HCC1937 and MDA-MB-468 cells. For overexpressing or silencing miR-520d-5p, miR-520d-5p mimics or inhibitor was transfected into HCC1937 and MDA-MB-468 cells with NC mimics or inhibitor as a negative control, separately. All above plasmids or oligonucleotides were purchased from GenePharma and were transfected into HCC1937 and MDA-MB-468 cells using Lipofectamine 2000 (Invitrogen) for 2 days.

### RT-qPCR analysis

Using TRIzol Reagent (Invitrogen), total RNA was extracted from MCF10A, HCC1937, MDA-MB-468, MDA-MB-231, MDA-MB-436 cells and then reverse-transcribed into cDNA with a Reverse Transcription Kit (Takara). A One Step TB Green^®^ PrimeScript™ RT-PCR Kit (Takara) was utilized for RT-qPCR on a Bio-Rad CFX96 Real-Time PCR system. The 2^−∆∆Ct^ method [22] was used for calculation of relative expression fold changes. GAPDH served as the internal reference for PITPNA-AS1 and mRNAs, while U6 served as the internal reference for miRNAs. The thermo-cycling conditions were set as follows: 95 °C for 5 min followed by 45 cycles at 95 °C for 10 s and 55 °C for 30 s, and a melting curve analysis every 0.2 °C from 55 to 95 °C for 2 min was obtained. Relative primer sequences are listed in Table 1, which were designed using GETprime software and synthesized by RiboBio (Guangzhou, China).

**Table 1:** Relative primer sequences used for PCR

Gene	Primer sequences
PITPNA-AS1	Forward: 5′-GCAGGGTGGATAAAGAGGA-3′
	Reverse: 5′-CCTACTGACAGGATGTCCT-3′
miR-524-5p	Forward: 5′-CTACAAAGGGAAGCACTTTCTCG-3′
	Reverse: 5′-CTCTACAGCTATATTGCCAGCCAC-3′
miR-520d-5p	Forward: 5′-CTACAAAGGGAAGCCCTTTCG-3′
	Reverse: 5′-CTCTACAGCTATATTGCCAGCCAC-3′
U6	Forward: 5′-CTCGCTTCGGCAGCACA-3′
	Reverse: 5′-AACGCTTCACGAATTTGCGT-3′
DENND1B	Forward: 5′-TGCATCCTTAGTTACCTTCCC-3′
	Reverse: 5′-CAGTTCCTTAGCCAAGTAATCTG-3′
ATL3	Forward: 5’-CAAGAGGAGCAGTGTAATGTG-3′
	Reverse: 5′-AAACGTTCCATTGCTTCGT-3′
SIK2	Forward: 5′-GAGGTCCACAACAGGTCTC-3′
	Reverse: 5′-TGCTACAATTCCCTGGGTG-3′
SNRNP200	Forward: 5′-CTCCAATGCCAAGGATGTG-3′
	Reverse: 5′-CTGATGTTGAAGCCCTGGA-3′
AFF3	Forward: 5′-TACAACTTCCACACCAGCA-3′
	Reverse: 5′-ATCTCCAAGCAAGGTCCTG-3′
UBE2J1	Forward: 5′-GTACATCGTACGGACTCCAG-3′
	Reverse: 5′-TCATGGAGGTATTCTTAGCTACAG-3′
DECR1	Forward: 5′-GGTAAAGGAATGACAACTCTTCTG-3′
	Reverse: 5′-CATTTCGGCTGGCTATCAC-3′
DDX54	Forward: 5′-TGGTGAGTGATCGAGTGAG-3′
	Reverse: 5′-CTTCCTGTCCTGACTGTCC-3′
MYBL2	Forward: 5′-TCCTGGATTCCTGTAACAGC-3′
	Reverse: 5′-AGTTCAGAAACTGGGAGGG-3′
E2F6	Forward: 5ʹ-TAGATGGATAGGATCTGATCTTAGC-3ʹ
	Reverse: 5ʹ-GATAAGTCAGAAAGTTCCTCCTG-3ʹ
PAX5	Forward: 5ʹ-TGTTTGCCTGGGAGATCAG-3ʹ
	Reverse: 5ʹ-CGGATGATCCTGTTGATGGA-3ʹ
FOXP2	Forward: 5ʹ-GATGCAACAACTCCAGCAG-3ʹ
	Reverse: 5ʹ-AGGACTTAAGCCAGCTTGAG-3ʹ
ELK4	Forward: 5ʹ-GCCCACTGGGAATACTGAG-3ʹ
	Reverse: 5ʹ-TCAGTATGATGGGTGTCTGTG-3ʹ
YY1	Forward: 5ʹ-GAATTTGCTAGGGCTGCAC-3ʹ
	Reverse: 5ʹ-CACATTCTGCACAGACGTG-3ʹ
GAPDH	Forward: 5ʹ-GCATCCTGGGCTACACTG-3ʹ
	Reverse: 5ʹ-TGGTCGTTGAGGGCAAT-3ʹ

### Subcellular fractionation assay

Subcellular fractionation assay was conducted to assess subcellular localization of PITPNA-AS1. According to manufacturer’ instructions, nuclear and cytoplasmic fractions extracted from TNBC cells were isolated using a Cytoplasmic and Nuclear RNA Purification Kit (Norgen). RT-qPCR analysis was applied for the detection of PITPNA-AS1, GAPDH or U6 expression. GAPDH was a cytoplasmic control and U6 was a nuclear control.

### Fluorescence in situ hybridization (FISH) assay

FISH assay was conducted for detection of the subcellular location of PITPNA-AS1. HCC1937 and MDA-MB-468 cells (5 × 10^3^ cells per well) were cultured in a 24-well plate, and the supernatant was discarded after 24 h. After being washed with PBS and fixed by 4% paraformaldehyde, the cells were permeabilized with PBS containing 0.5% Triton X-100. Next, the cells were blocked with the pre-hybridization solution for 4 h at 37 °C and hybridized with PITPNA-AS1 specific probe (RiboBio) overnight at 37 °C followed by washing with the hybridization solution at 42 °C in the dark. Afterwards, 4′-6-diamidino-2-phenylindole (DAPI) was utilized to stain nucleus for 10 min, and a fluorescence microscope (Olympus) was used to capture the images of the cells.

### Cell counting kit-8 (CCK-8) assay

Based on manufacturer’s requirements, cell viability was testified by a CCK-8 kit (Boster Biological Technology, CA, USA). In brief, transfected HCC1937 and MDA-MB-468 cells were plated into 96-well plates at a density of 2 × 10^3^ cells/well and were incubated for 0, 24, 48 and 72 h. Next, 10 μL of CCK-8 solution was added into each well for further incubation in 5% CO_2_ at 37 °C for 1 h after cell adhesion. The culture medium was then removed, and the plates were washed twice by PBS. Lastly, absorbance at 450 nm was monitored by a microplate reader (EL340; Bio-Tek Instruments, Hopkinton, MA, USA) for detecting cell viability.

### Colony formation assay

Colony formation assay was performed to reveal cell proliferation. In brief, 1 × 10^3^ HCC1937 and MDA-MB-468 cells were seeded in 6-well plates. At 2 weeks after incubation, colonies were fixed by methanol for 15 min and were dyed by crystal violet (Sigma-Aldrich) in PBS for 20 min. After that, crystal violet was slowly washed away with running water. The plates were air-dried in an inverted position and number of stained colonies were manually counted.

### Flow cytometry analysis

Transfected HCC1937 and MDA-MB-468 cells were subjected to the staining of propidium iodide (PI) and FITC-Annexin V for detection of apoptosis rate. HCC1937 and MDA-MB-468 cells were seeded in 6-well plates at a concentration of 1 × 10^5^ cells/well, after which cells were incubated in 5 μL of Annexin V-FITC for 10 min and 10 μL of PI for 15 min at 4 °C in the dark. Later, using CellQuest software (BD Biosciences, San Jose, CA), cells were analyzed by flow cytometer (FACScan; BD Biosciences), and apoptosis rate of TNBC cells was assessed. The early apoptotic cells were distributed in the second quadrant and late apoptotic and necrotic cells were in the third and fourth quadrants. Apoptosis rate (%) was defined as cell percentage in the third quadrant.

### Wound healing assay

Wound healing assay was performed to detect cell migration. TNBC cells (1.5 × 10^6^) were plated into the 6-well plates. Then, a pipette tip was utilized to horizontally scratch the wound after cell reached about 80% confluence. After cells were washed twice, cells were incubated in serum-free medium for 24 h. The gap between cells was photographed under a low-magnification phase-contrast microscope (Olympus MK, Tokyo, Japan).

### Transwell assay

Transwell assay was conducted to reveal cell migration in vitro. Matrigel-coated upper chamber (8.0 μm pore size, BD Biosciences) was filled with TNBC cells (5 × 10^4^/well) suspended in DMEM. The DMEM supplemented by 10% FBS was placed in the bottom chamber. Forty-eight hours later, cells in the upper chamber were scraped off by cotton swabs. Cells in the lower chamber were fixed with methanol and stained with 0.5% crystal violet. Finally, the average number of stained cells were counted using an inverted microscope (Olympus) under five randomly selected visual fields by Image J software.

### Western blot analysis

RIPA lysis buffer containing protease inhibitors was used for the extraction of total protein from HCC1937 and MDA-MB-468 cells. Before being transferred to a polyvinylidene fluoride membrane (Millipore), equivalent proteins were isolated by 10% sodium dodecyl sulfate polyacrylamide gel electrophoresis. After being blocked with 5% skimmed milk for 1 h, the membranes were incubated with primary antibodies against E-cadherin (ab40772, 1/20000), N-cadherin (ab76011, 1/5000), Vimentin (ab92547, 1/200), Slug (ab27568, 1/500), Twist (ab175430, 1/1000), SIK2 (ab53423, 1/1000), DDX54 (ab76947, 1/2000), and GAPDH (ab9485, 1/2500) overnight at 4 °C. Next, secondary antibody (ab6721, 1/10000) conjugated with HRP was added for further incubation of 1 h at room temperature. All the antibodies were purchased from Abcam (Cambridge, USA). A chemiluminescence detection system was applied to visualize the proteins. The gray value of each protein band was analyzed by the ImageJ software, and the ratio of the gray value of the target protein to GAPDH was calculated.

### In vivo experiment

For animal study, a total of 20 female BALB/C nude mice (6-week-old; weighing 20–30 g) were purchased from Shi Laike Company. The animal experiments were performed with ethical approval from the Second Hospital of Shanxi Medical University (Shanxi, China). The nude mice were subcutaneously injected with 0.2 mL suspension of HCC1937 cells (3 × 10^6^) that were stably transfected with sh-PITPNA-AS1 or sh-NC. The tumor volume was measured every 4 days. ${\text{Volume}}\,({\text{mm}}^{3} ) = 1/2\,{\text{L}} \times {\text{D}}^{2} .$ L represents the maximum diameter, and D represents the shortest diameter of the tumor. After 28 days, mice were euthanized, and tumors were excised for weighing.

### Immunohistochemistry staining (IHC)

Tumor tissues were fixed in 4% paraformaldehyde, and then dehydrated in ethanol solutions. Next, tissues were embedded in paraffin, and cut into 4 μm sections. The sections were routinely deparaffinized, followed by antigen retrieval. After washing in PBS for 15 min, tissues were blocked with goat serum for 1 h followed by incubation with primary antibody against Ki67 (ab92742, 1/500) overnight at 4 °C. Subsequently, the sections were cultivated with HRP-conjugated secondary antibody (ab6721, 1/1000). The slides were then counterstained with hematoxylin for 1 min and mounted with neutral balsam. The Ki67 positivity was detected using a Histostain™ SP-9000 immunohistochemical staining kit (Zymed Laboratories, South San Francisco, CA, USA). An OLYMPUSBX-41 microscope (Olympus) was used to capture the images.

### RNA pull down assay

HCC1937 and MDA-MB-468 cells were transfected with 50 nM biotin-labeled PITPNA-AS1 for 48 h. Cells were then incubated in RIPA lysis buffer for 10 min and then the mixture were centrifuged at 14,000×*g* to obtain the supernatant. Protein lysate was incubated with M-280 streptavidin beads which were pre-coated with RNase-free bovine serum albumin and yeast tRNA. Next, the beads were incubated at 4 °C for 3 h. The binding miR-520d-5p was purified by TRIzol and its expression was examined by RT-qPCR. As per manufacture’s guidelines, a Pierce Magnetic RNA–Protein Pull-Down Kit (Thermo Fisher Scientific) was employed to detect the proteins binding with PITPNA-AS1. Protein extracts from HCC1937 or MDA-MB-468 cells were subjected to biotinylated PITPNA-AS1 (50 pmol) and M-280 streptavidin magnetic beads (Invitrogen) for 1 h at 4 °C. Finally, the cells were washed five times with RIPA buffer, and incubated with 5× loading buffer at 95 °C for 5 min. The eluted DDX54, FMR1, IGF2BP1, IGF2BP2 proteins were detected by western blotting.

### Luciferase reporter assay

For luciferase reporter assay, PITPNA-AS1-WT/Mut or SIK2-WT/Mut reporter was constructed by subcloning wild-type (WT) or mutant (Mut) sequences of PITPNA-AS1 complementary to miR-520d-5p or SIK2 3′UTR into the pmirGLO dual-luciferase vector. Then, these constructed reporters were co-transfected with miR-520d-5p mimics or NC mimics into HCC1937 or MDA-MB-468 cells. For PITPNA-AS1 promoter-luciferase analysis, wild type or mutated PITPNA-AS1 promoter sequences (site 1 and site 2) were subcloned into the pGL3-Basis luciferase vector (Promega, Madison, WI, USA), and these constructs were co-transfected with pcDNA3.1/MYBL2 or pcDNA3.1 into HCC1937 and MDA-MB-468 cells. After 2 days, a Dual-Luciferase Report Assay System (Promega) was used for the measurement of luciferase activity. Relative luciferase activity was defined as the ratio of the relative light unit of firefly luciferase to that of *Renilla* luciferase.

### RNA immunoprecipitation (RIP) assay

Based on the manufacturer’s instruments, an EZ-Magna RIP kit (Millipore) was applied. In brief, HCC1937 and MDA-MB-468 cells were washed with pre-cooled PBS and then lysed in RIPA lysis buffer in an ice bath for 5 min, and centrifuged at 12,000×*g* for 10 min at 4 °C. Then, cell extract was incubated with magnetic beads coated human anti-Ago2 (Millipore), anti-DDX54 (Millipore) or control anti-IgG (Millipore) at 4 °C overnight. Finally, expression of PITPNA-AS1, miR-520d-5p, SIK2 immunoprecipitated by Ago2 and expression of PITPNA-AS1, SIK2 immunoprecipitated by DDX52 were analyzed by RT-qPCR after being purified by proteinase K.

### Actinomycin D assay

Actinomycin D can inhibit the synthesis of mRNAs. After transfection with sh-NC, sh-PITPNA-AS1#1, sh-DDX54#1 for 48 h, HCC1937 and MDA-MB-468 cells were treated with actinomycin D (5 μmol/L) for 0, 4, 8, 12 h. Later, RT-qPCR was used for the evaluation of relative SIK2 expression levels.

### Chromatin immunoprecipitation (ChIP) assay

With a Magna ChIP Kit (Millipore), ChIP was performed to explore which site is responsible for the binding between MYBL2 and PITPNA-AS1 promoter. Briefly, crosslinked chromatin DNA was separated into fragments of 200–2000 bp through sonication. Next, lysates were immunoprecipitated with anti-MYBL2 or anti-IgG (internal control) at 4 °C overnight. Subsequently, the mixture was centrifuged, and the precipitate was washed with the low salt buffer, the high salt buffer, the LiCl solution, and the trace element solution. The protein-DNA complex was eluted with 250 μL of ChIP Wash Buffer and de-crosslinked with 20 μL of 5 M NaCl. Quantity of immunoprecipitated DNA was detected by RT-qPCR.

### Statistical analysis

Data were expressed as mean ± standard deviation and were analyzed by SPSS 22.0 (SPSS, Chicago, USA) from three biological and technical replications. The variance significance was evaluated by Student’s *t* test for difference between two group or ANOVA for that among three groups. P < 0.05 was set as the threshold of statistical significance.

## Results

### PITPNA-AS1 was upregulated in TNBC and localized in the cytoplasm

**Fig. 1:**
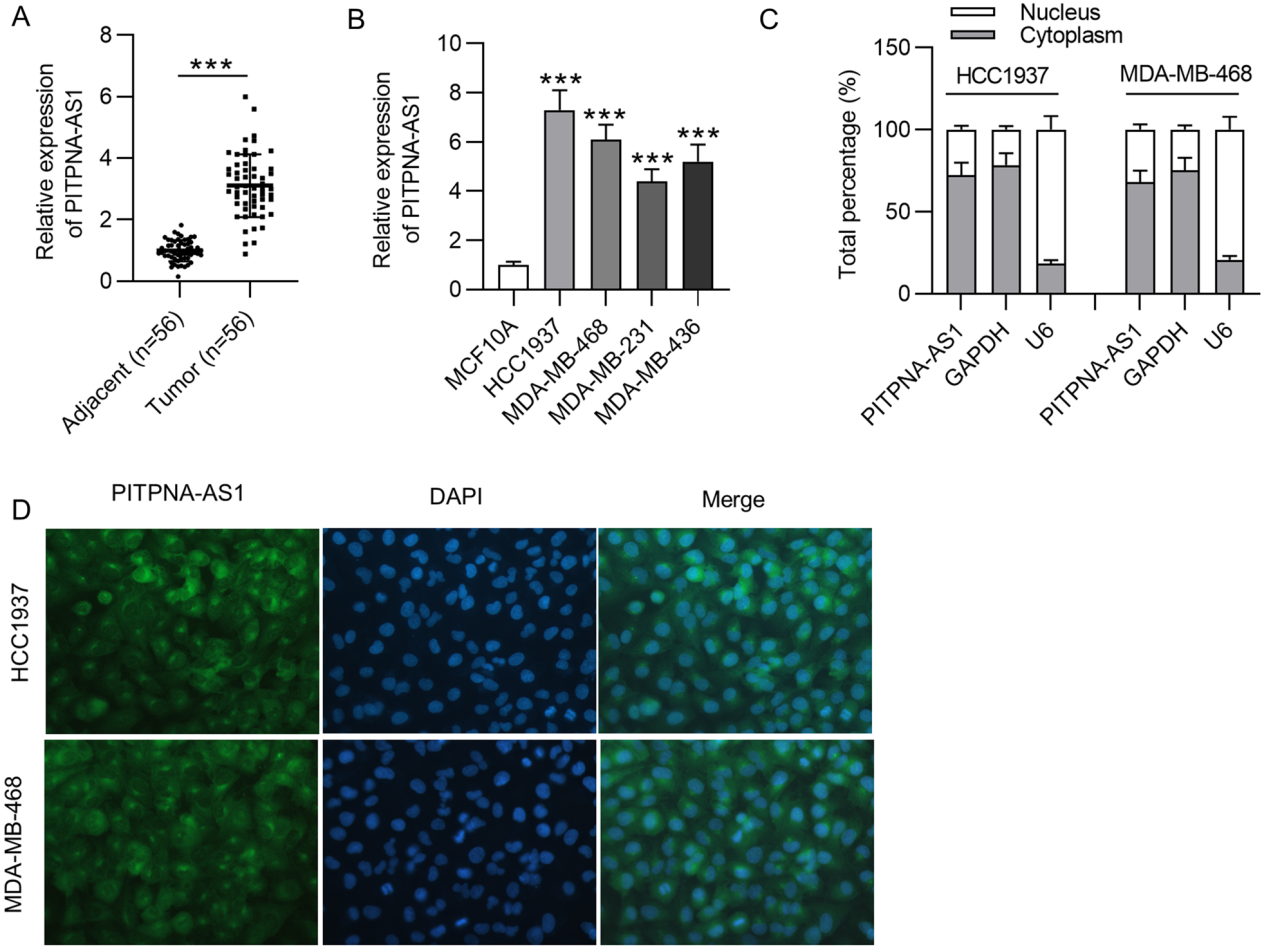
The expression and localization of PITPNA-AS1 in TNBC cells. **A** RT-qPCR analysis of PITPNA-AS1 expression in TNBC tissues and matched non-tumor tissues. **B** PITPNA-AS1 expression in TNBC cell lines and MCF10A cell lines was examined by RT-qPCR. **C**, **D** Subcellular localization of PITPNA-AS1 was assessed via subcellular fractionation and FISH assays. ***p < 0.001

First, the expression pattern of PITPNA-AS1 in TNBC was detected by RT-qPCR. PITPNA-AS1 expression was significantly upregulated in TNBC tissues compared to that in adjacent nontumor tissues (Fig. 1A). Moreover, PITPNA-AS1 expression in TNBC cell lines (HCC1937, MDA-MB-468, MDA-MB-231 and MDA-MB-436) showed a higher level compared to that in MCF10A cell line (Fig. 1B). HCC1937 and MDA-MB-468 cells were used for the following assays since they contained the relatively higher expression of PITPNA-AS1. The results of subcellular fractionation assay manifested that PITPNA-AS1 was majorly distributed in the cytoplasm of HCC1937 and MDA-MB-468 cells (Fig. 1C). Furthermore, results of FISH assay confirmed PITPNA-AS1 as a cytoplasmic RNA in TNBC (Fig. 1D), indicating that PITPNA-AS1 might regulate gene expression at the post-transcriptional level.

### PITPNA-AS1 knockdown suppressed TNBC cellular process in vitro

**Fig. 2:**
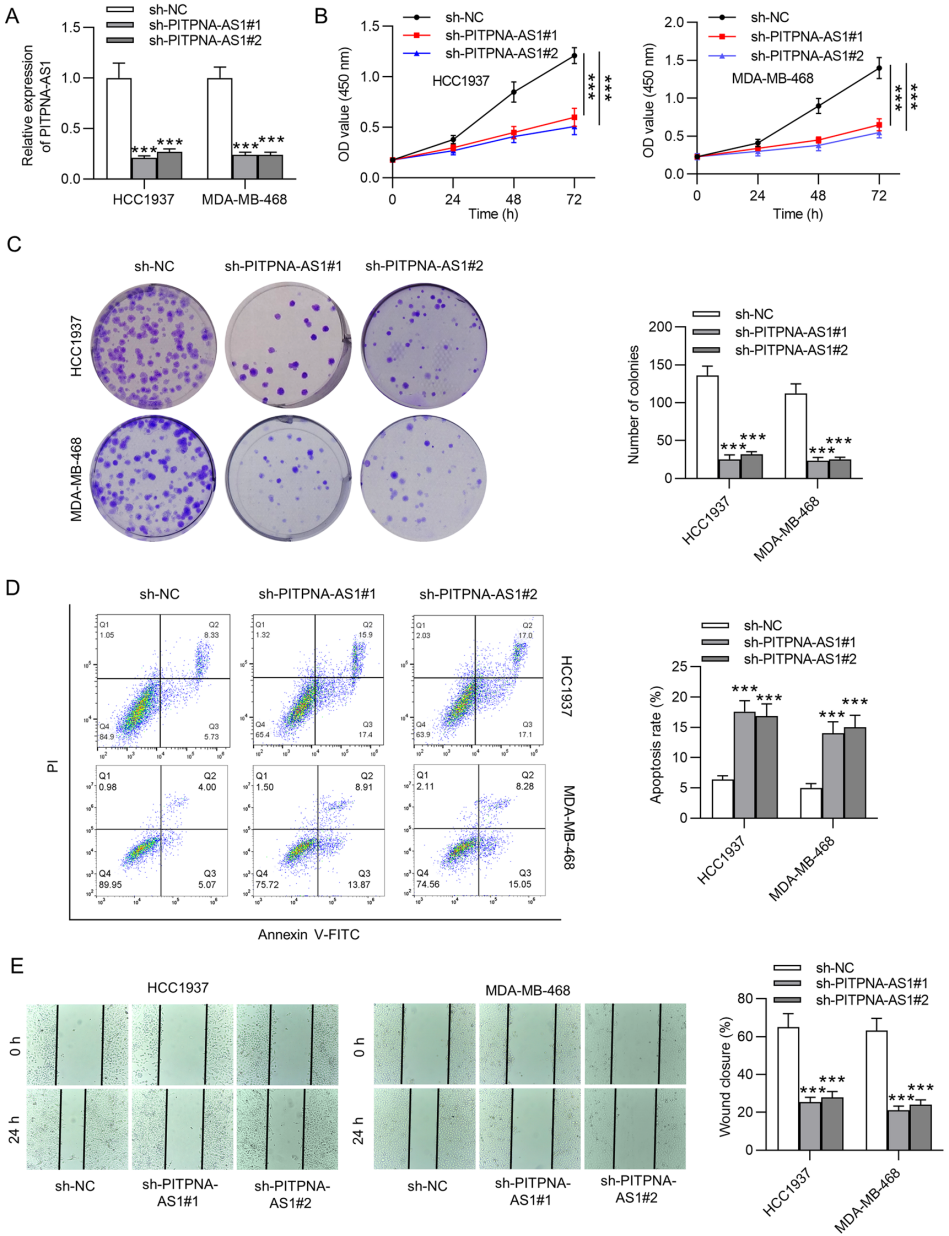

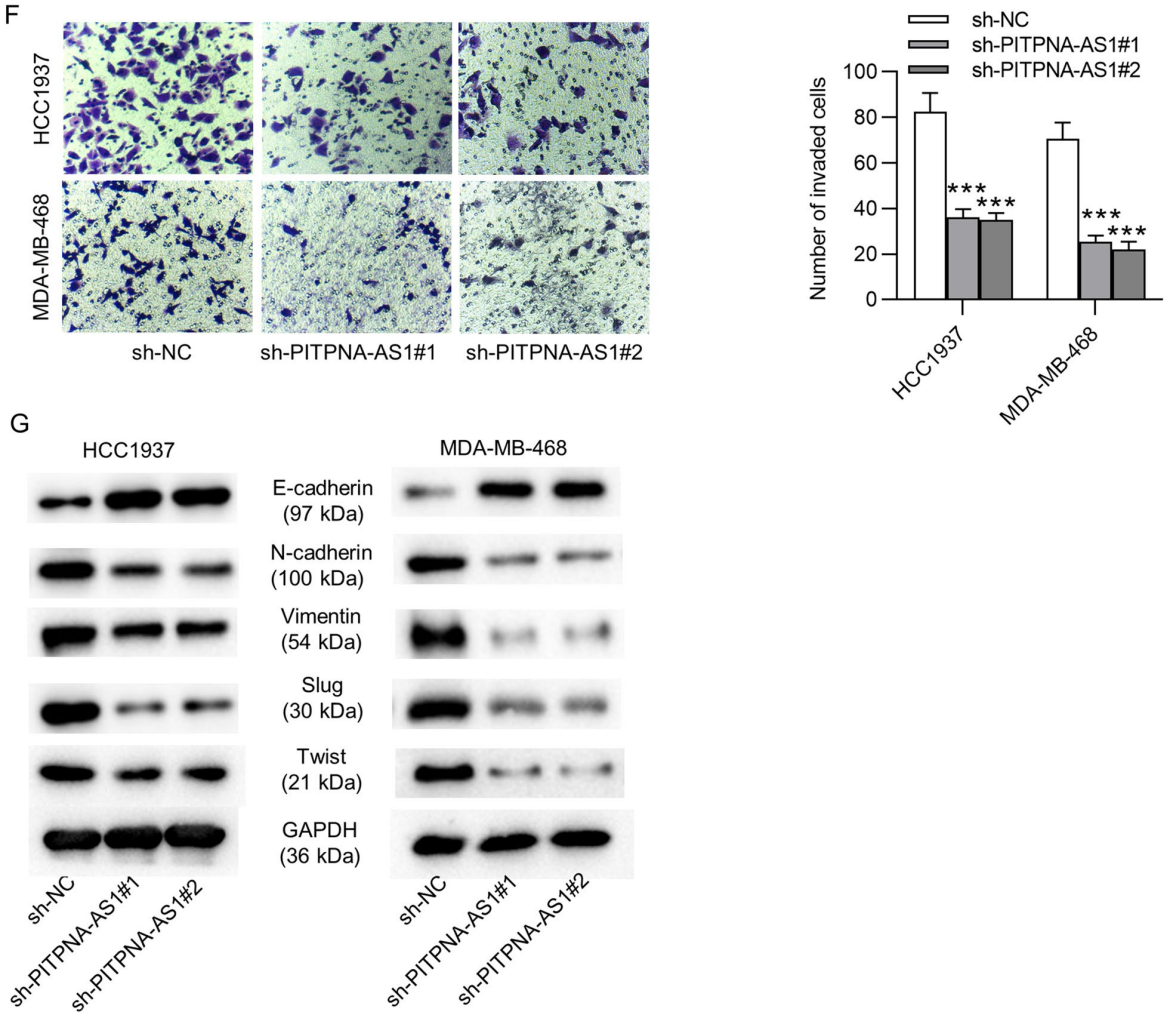
The biological function of silenced PITPNA-AS1 in vitro. **A** RT-qPCR analysis was conducted to verify PITPNA-AS1 knockdown efficiency in HCC1937 and MDA-MB-468 cells. **B**, **C** CCK-8 and colony formation assays evaluated the proliferative ability of HCC1937 and MDA-MB-468 cells transfected with sh-PITPNA-AS1#1/2 or sh-NC. **D** Flow cytometry analysis was conducted to detect the effect of PITPNA-AS1 knockdown on HCC1937 and MDA-MB-468 cell apoptosis. **E**, **F** Cell migration and invasion in PITPNA-AS1-silenced cells was analyzed by wound healing and Transwell assays. **G** Levels of proteins related to the EMT process were tested by western blot analysis in sh-PITPNA-AS1#1/2 or sh-NC group. ***p < 0.001

Considering the upregulation of PITPNA-AS1 in TNBC, we then explored the functional role of PITPNA-AS1 by loss-of-function assays. PITPNA-AS1 expression was stably silenced in HCC1937 and MDA-MB-468 cells by transfection of sh-PITPNA-AS1#1 and sh-PITPNA-AS1#2 (Fig. 2A). As showed in Fig. 2B, HCC1937 and MDA-MB-468 cell viability was suppressed by silencing PITPNA-AS1. Moreover, results of colony formation assay demonstrated that PITPNA-AS1 suppressed HCC1937 and MDA-MB-468 cell proliferation (Fig. 2C). Furthermore, decreased PITPNA-AS1 expression promoted the apoptosis rate of HCC1937 and MDA-MB-468 cells (Fig. 2D). Through results of the wound healing assay, we found that the migrative ability of HCC1937 and MDA-MB-468 cells was inhibited by PITPNA-AS1 knockdown (Fig. 2E). Furthermore, downregulated PITPNA-AS1 suppressed the invasive capability of HCC1937 and MDA-MB-468 cells (Fig. 2F). Similarly, PITPNA-AS1 deficiency induced increase in E-cadherin protein level and decrease in N-cadherin, Vimentin, Slug and Twist protein levels, revealing that the epithelial-mesenchymal transition (EMT) process was inhibited by silenced PITPNA-AS1 in HCC1937 and MDA-MB-468 cells (Fig. 2G).

### PITPNA-AS1 silencing inhibited tumor growth in vivo

**Fig. 3:**
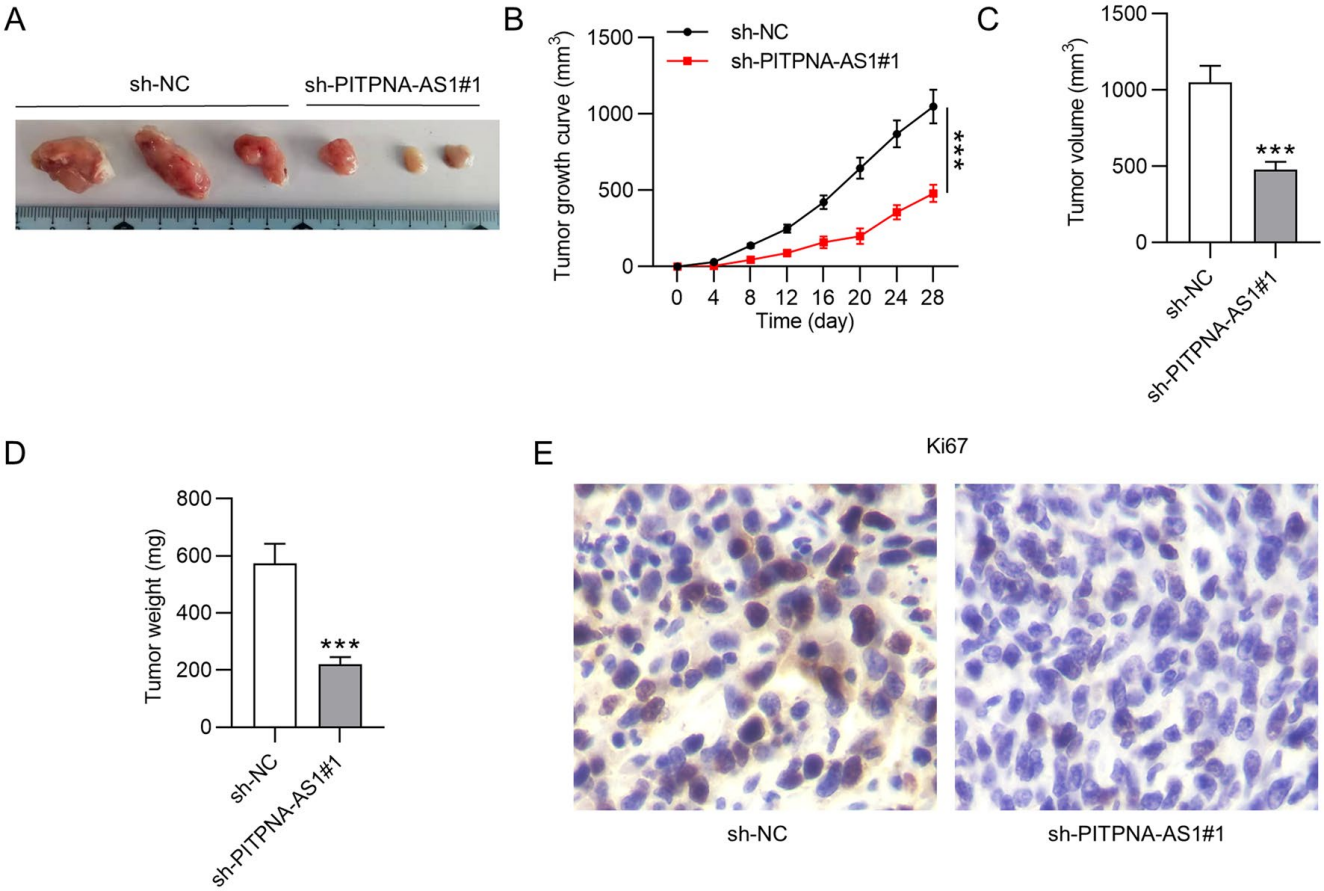
Knockdown of PITPNA-AS1 suppressed TNBC tumor growth. **A** Tumors excised from the nude mice in sh-PITPNA-AS1#1 and sh-NC group. **B**–**D** Tumor growth, volume and weight were analyzed after nude mice were subcutaneously injected with transfected HCC1937 cells. **E** Ki67 expression in tumors was shown by IHC assay after silencing PITPNA-AS1. ***p < 0.001

Later, the biological effect of PITPNA-AS1 on tumor growth in vivo was explored by animal experiments. At first, HCC1937 cells stably transfected with sh-PITPNA-AS1#1 or sh-NC were subcutaneously injected into nude mice. We found that tumor size in sh-PITPNA-AS1#1 group was smaller than that in sh-NC group (Fig. 3A). Tumor growth in sh-PITPNA-AS1#1 group was slower than that in sh-NC group (Fig. 3B). Furthermore, tumor volume and tumor weight were both reduced by PITPNA-AS1 knockdown (Fig. 3C, D). More importantly, results of IHC assay in xenograft tumors confirmed that PITPNA-AS1 depletion reduced expression of Ki67, suggesting that PITPNA-AS1 downregulation repressed cell proliferation in tumors (Fig. 3E).

### PITPNA-AS1 sequestered miR-520d-5p in TNBC cells

**Fig. 4:**
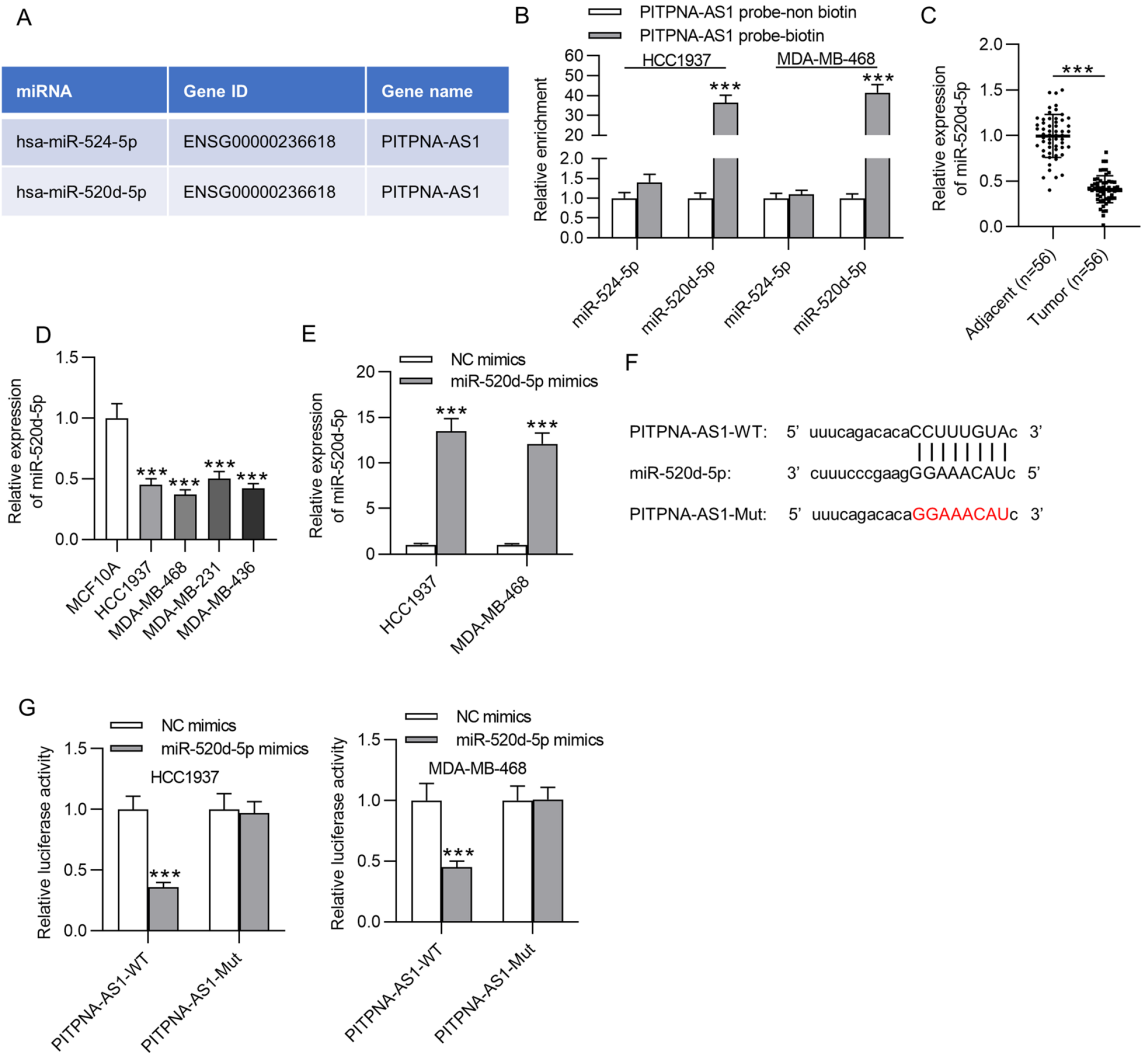
PITPNA-AS1 acted as a sponge of miR-520d-5p. **A** Potential miRNAs for PITPNA-AS1 were obtained from starBase. **B** The binding capacity between PITPNA-AS1 and predicted miRNAs was assessed by RNA pull-down assay. **C** MiR-520d-5p expression in TNBC samples and adjacent non-tumor specimens was detected through RT-qPCR analysis. **D** RT-qPCR was utilized for evaluating miR-520d-5p expression in TNBC cell lines and MCF10A cell line. **E** MiR-520d-5p expression in HCC1937 and MDA-MB-468 cell transfected with miR-520d-5p mimics by RT-qPCR. **F** A binding site between PITPNA-AS1 and miR-520d-5p was demonstrated. **G** Luciferase reporter assay validated the binding of PITPNA-AS1 to miR-520d-5p. ***p < 0.001

Based on the post-transcriptionally regulatory role of PITPNA-AS1 in TNBC cells, we speculated that PITPNA-AS1 might sponge specific miRNA to regulate TNBC progression through the ceRNA network. We searched the potential miRNAs for PITPNA-AS1 using starBase (http://starbase.sysu.edu.cn/). Under the condition of CLIP data ≥ 3, Degradome Data ≥ 3, two putative miRNAs (miR-524-5p and miR-520d-5p) were discovered (Fig. 4A). Further, RNA pull-down assay showed high enrichment of miR-520d-5p in PITPNA-AS1 biotin probe group, while miR-524-5p was not significantly enriched by the PITPNA-AS1 biotin probe (Fig. 4B), indicating the potential binding of PITPNA-AS1 to miR-520d-5p but not miR-524-5p. Later, a remarkable downregulation of miR-520d-5p was detected in TNBC tissues by RT-qPCR (Fig. 4C). Furthermore, low expression level of miR-520d-5p was also observed in TNBC cell lines (Fig. 4D). Subsequently, miR-520d-5p mimics was transfected into HCC1937 and MDA-MB-468 cells to overexpress miR-520d-5p for further analysis (Fig. 4E). In addition, a complementary binding sequence between PITPNA-AS1 and miR-520d-5p was revealed by starBase (Fig. 4F). To confirm the interaction of PITPNA-AS1 and miR-520d-5p, luciferase reporter assay was conducted. The results revealed the weakened luciferase activity of PITPNA-AS1-WT induced by miR-520d-5p upregulation. No significant difference was found in luciferase activity of PITPNA-AS1-Mut in HCC1937 and MDA-MB-468 cells after transfection of miR-520d-5p mimics compared to after transfection of NC mimics (Fig. 4G).

### SIK2 was the target gene of miR-520d-5p in TNBC cells

**Fig. 5:**
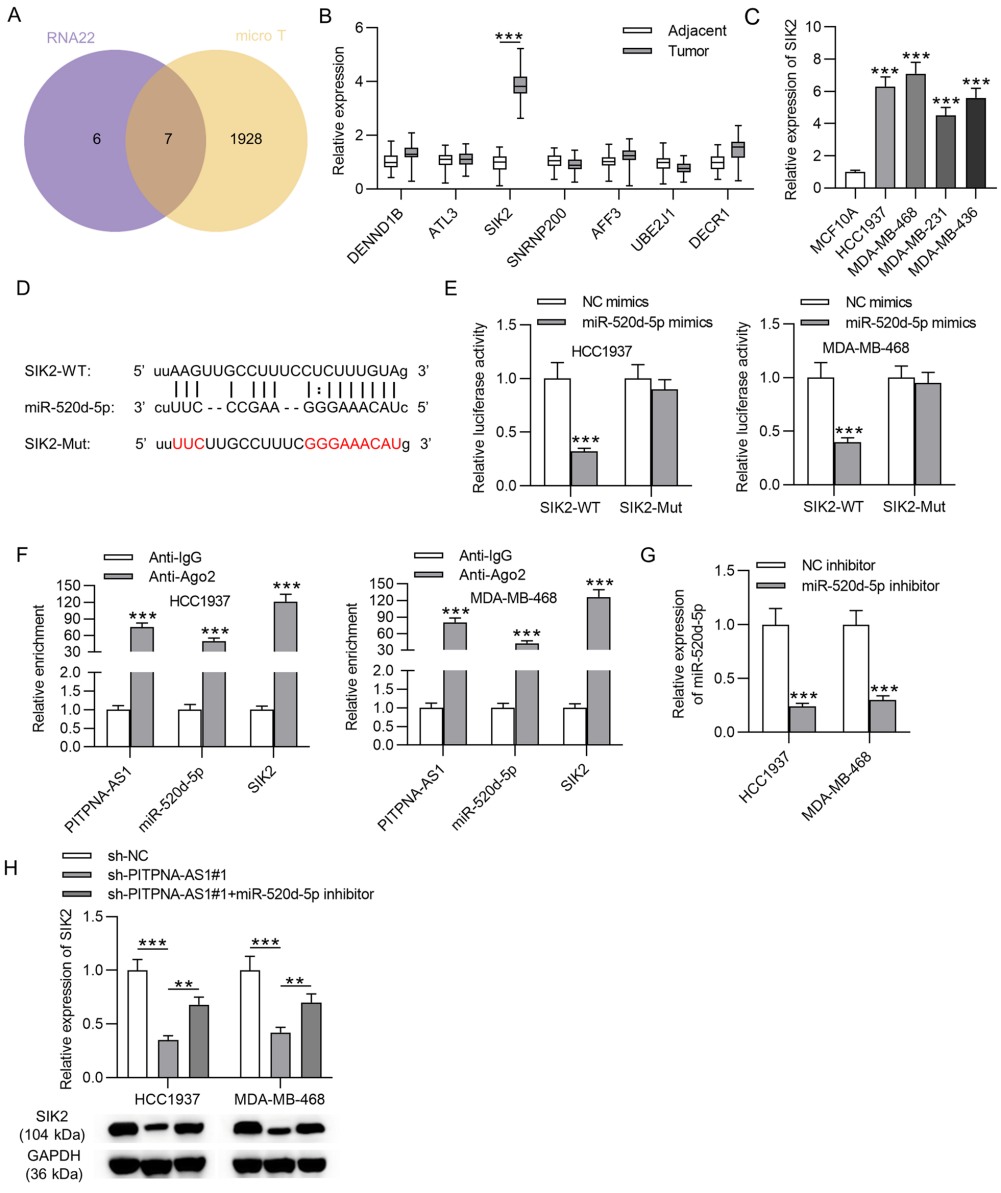
MiR-520d-5p targeted SIK2 in TNBC. **A** Target mRNAs for miR-520d-5p based on prediction of DIANA and microT databases. **B** Expression levels of predicted mRNAs in TNBC tissues and adjacent non-tumor tissues were determined by RT-qPCR. **C** SIK2 expression in TNBC cells and MCF10A cell line was detected via RT-qPCR. **D** The binding sequence of miR-520d-5p on SIK2 3′UTR was predicted through starBase. **E** The binding of miR-520d-5p to SIK2 was confirmed by luciferase reporter assay. **F** RIP assay showed the enrichments of PITPNA-AS1, miR-520d-5p and SIK2 in the beads-conjugated with anti-IgG or anti-Ago2. **G** The efficiency of miR-520d-5p inhibition in HCC1937 and MDA-MB-468 cells was examined by RT-qPCR. **H** SIK2 expression in HCC1937 and MDA-MB-468 cells in each group was detected by RT-qPCR and western blotting analyses. **p < 0.01, ***p < 0.001

To further support hypothesis of PITPNA-AS1 mediated ceRNA mechanism, miR-520d-5p target genes in TNBC were explored. Combining results from two online tools (RNA22 and microT), seven mRNAs were predicted as the downstream genes of miR-520d-5p (Fig. 5A). Expression of these mRNAs in TNBC tissues was testified by RT-qPCR analysis and only SIK2 was discovered to be prominently upregulated in TNBC tissues (Fig. 5B). Subsequently, SIK2 expression was higher in TNBC cell lines in comparison with that in MCF10A cell line (Fig. 5C). Afterwards, starBase predicted a binding site between miR-520d-5p and SIK2 (Fig. 5D). Luciferase reporter assay manifested that miR-520d-5p predominantly suppressed the luciferase activity of SIK2-WT but not that of SIK2-Mut (Fig. 5E). Moreover, PITPNA-AS1, miR-520d-5p and SIK2 were validated to co-exist in RNA-induced silencing complexes (RISCs) by RIP assay (Fig. 5F). Additionally, miR-520d-5p expression was inhibited in HCC1937 and MDA-MB-468 cells with transfection of miR-520d-5p inhibitor (Fig. 5G). We found that decreased mRNA and protein expression of SIK2 by PITPNA-AS1 knockdown was partly reversed by miR-520d-5p downregulation, suggesting that, in addition to miR-520d-5p, PITPNA-AS1 can regulate SIK2 expression by other molecules (Fig. 5H).

### PITPNA-AS1 recruited DDX54 protein to stabilize SIK2 mRNA

**Fig. 6:**
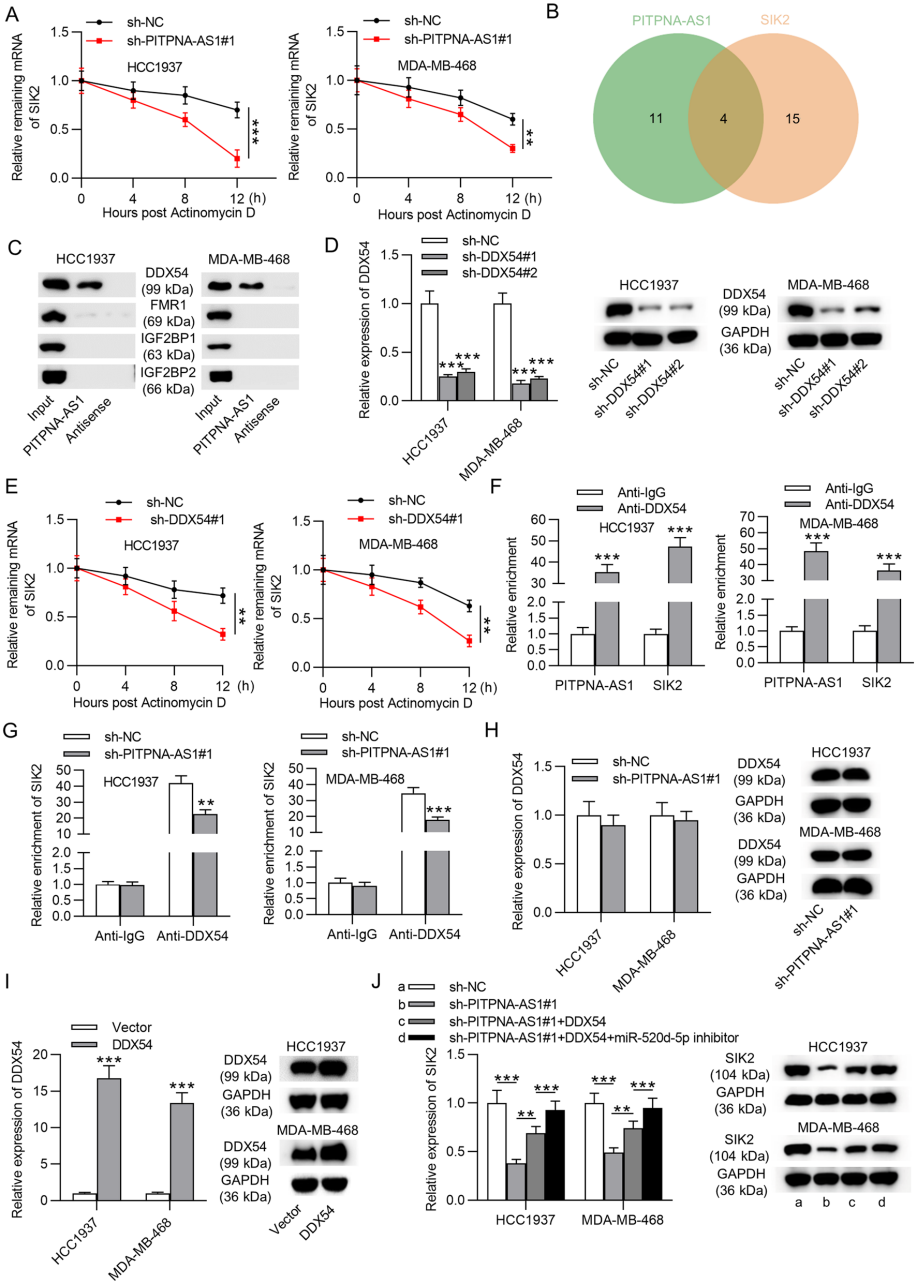
PITPNA-AS1 increased SIK2 mRNA stability via recruiting DDX54. **A** SIK2 mRNA stability in HCC1937 and MDA-MB-468 cells transfected sh-PITPNA-AS1#1 or sh-NC after treating with Actinomycin D was detected by RT-qPCR. **B** Four common RBPs for PITPNA-AS1 (CLIP Data ≥ 2) and SIK2 (CLIP Data ≥ 5) were predicted by starBase. **C** The binding capacity of PITPNA-AS1 to DDX54, FMR1, IGF2BP1 or IGF2BP2 was detected by RNA pull down assay. **D** Efficiency of silencing of DDX54 in HCC1937 and MDA-MB-468 cells was identified via RT-qPCR and western blot. **E** Stability of SIK2 mRNA in transfected cells treated with actinomycin D for 0, 4, 8, 12 h was evaluated by RT-qPCR. **F** The interaction of DDX54 protein with PITPNA-AS1 or SIK2 mRNA was verified through RIP assay. **G** RIP assay demonstrated the changes in the binding of DDX54 protein to SIK2 mRNA upon PITPNA-AS1 knockdown. **H** DDX54 expression at the mRNA and protein levels after silencing PITPNA-AS1 was measured by RT-qPCR and western blot. I RT-qPCR and western blot verified the overexpression efficiency of DDX54. **J** RT-qPCR and western blot analyses of SIK2 expression in HCC1937 and MDA-MB-468 cells transfected with indicated plasmids. **p < 0.01, ***p < 0.001

There was another post-transcriptional regulation of lncRNA maintaining mRNA stability by recruiting RBPs [19]. Based on above findings, we hypothesized that PITPNA-AS1 might modulate SIK2 expression not only by serving as a ceRNA but also by interacting with RBPs. SIK2 mRNA expression in TNBC cells treated with actinomycin D was detected by RT-qPCR. Results implied that PITPNA-AS1 knockdown inhibited SIK2 mRNA stability compared with sh-NC group (Fig. 6A). Subsequently, the potential RBPs that could interact with PITPNA-AS1 and SIK2 were explored. Through starBase, 4 RBPs (DDX54, FMR1, IGF2BP1 and IGF2BP2) were found (Fig. 6B). To narrow the selection, RNA pull-down assay was conducted, and the results suggested that only DDX54 could bind to PITPNA-AS1 in HCC1937 and MDA-MB-468 cells (Fig. 6C). Subsequently, to testify whether DDX54 exerted function on SIK2 mRNA stability, we knocked down DDX54 in HCC1937 and MDA-MB-468 cells with transfection of sh-DDX54#1/2, denoting sh-DDX54#1 with better knockdown efficiency (Fig. 6D). SIK2 mRNA stability was significantly decreased by DDX54 downregulation in HCC1937 and MDA-MB-468 cells treated with actinomycin D (Fig. 6E). Later, results of RIP assay further confirmed that DDX54 protein combined with PITPNA-AS1 and SIK2 (Fig. 6F). In addition, PITPNA-AS1 silencing weakened the interaction between DDX54 protein and SIK2 mRNA (Fig. 6G). Results of DDX54 mRNA and protein levels remained unchanged in sh-PITPNA-AS1#1 transfected TNBC cells, suggesting that PITPNA-AS1 recruited DDX54 to maintain SIK2 mRNA rather than regulating DDX54 expression (Fig. 6H). DDX54 overexpression efficiency was confirmed by RT-qPCR and western blotting (Fig. 6I). As shown in Fig. 6J, inhibited SIK2 mRNA and protein expression by silenced PITPNA-AS1 was partly countervailed by overexpressed DDX54, whereas was fully recovered by DDX54 upregulation and miR-520d-5p inhibition.

### PITPNA-AS1 upregulated SIK2 to play an oncogenic role in TNBC cells by miR-520d-5p and DDX54

**Fig. 7:**
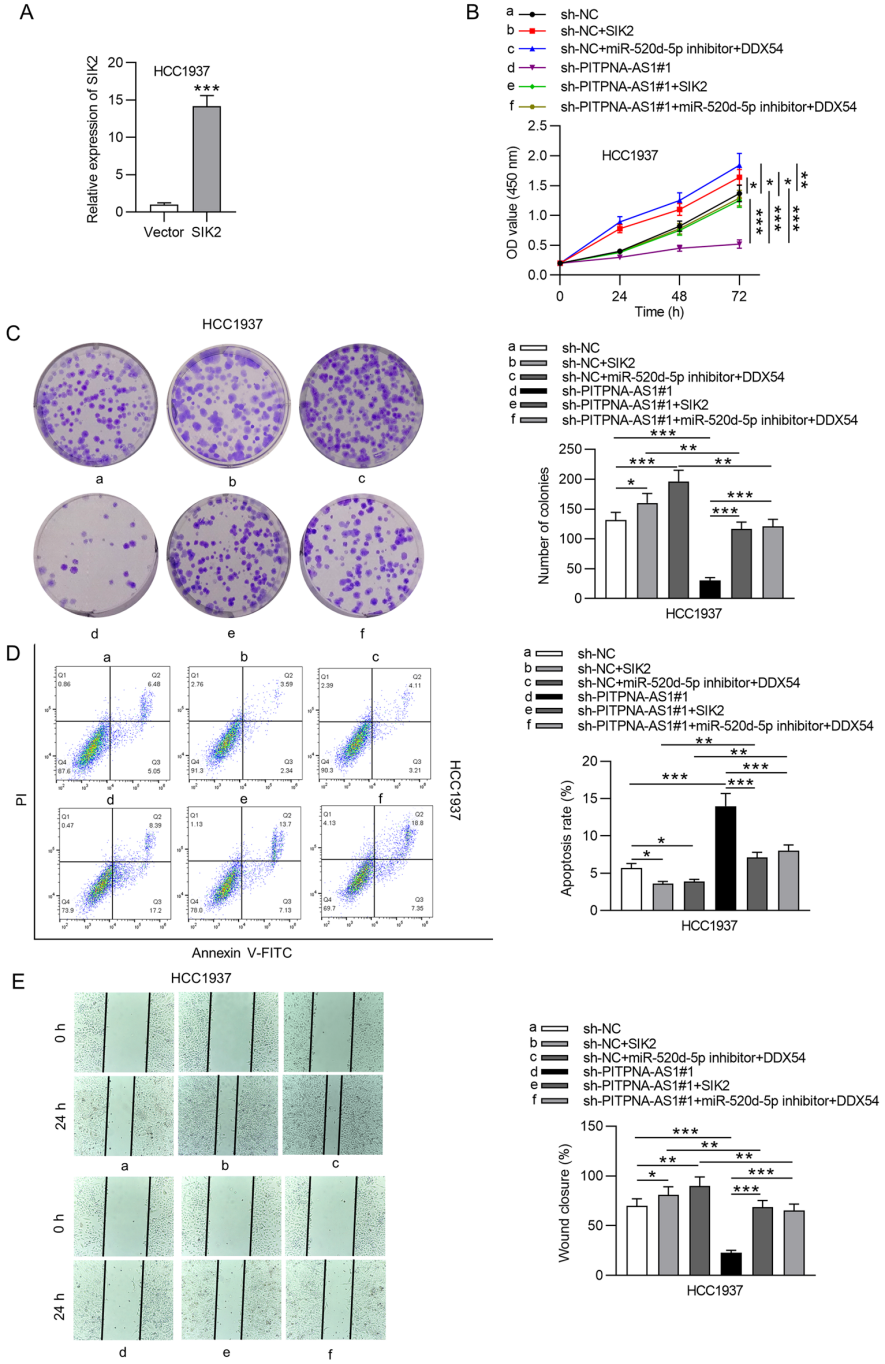

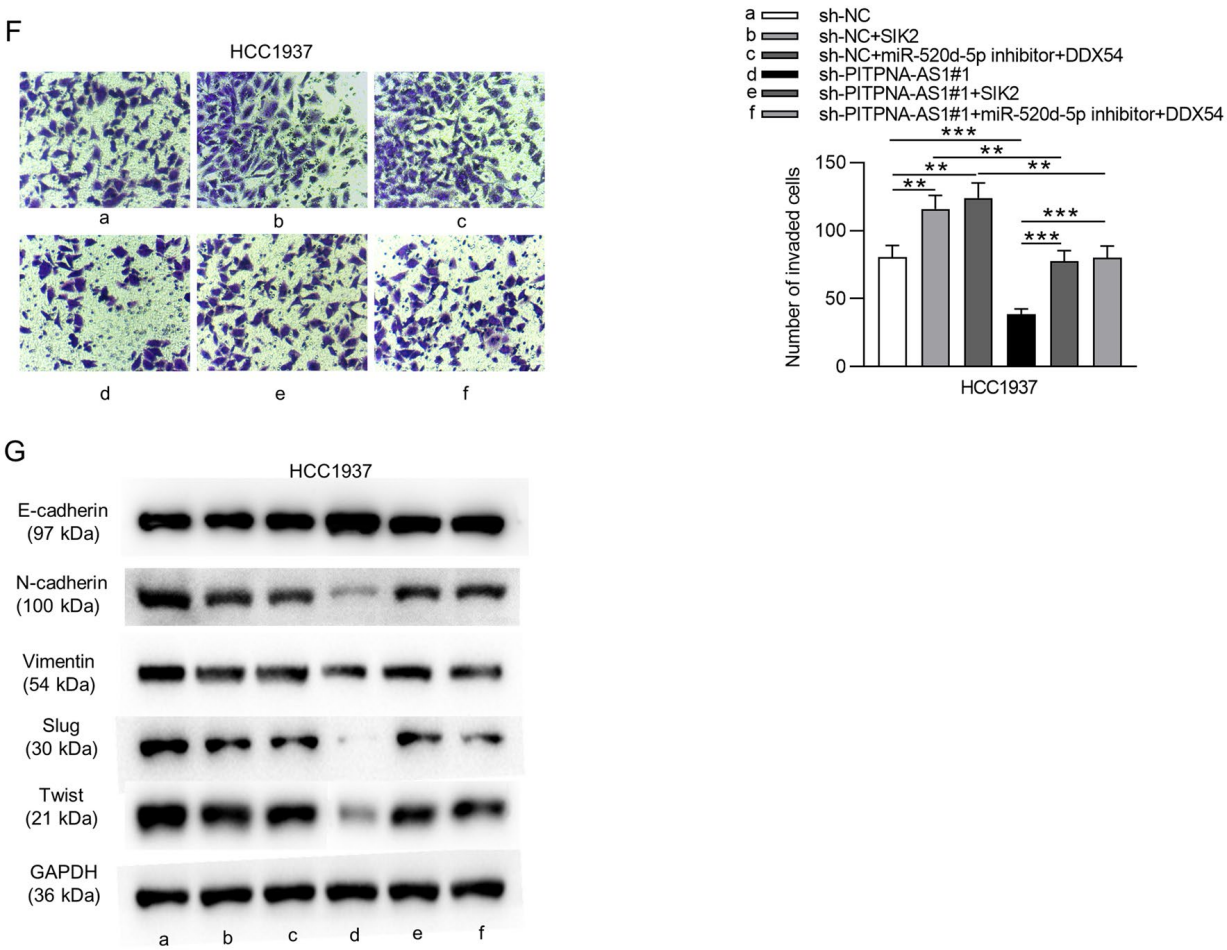
PITPNA-AS1 promoted TNBC cellular processes by targeting the miR-520d-5p/DDX54/SIK2 axis. **A** SIK2 expression in HCC1937 cell lines transfected with pcDNA3.1 (Vector) or pcDNA3.1/SIK2 (SIK2) was examined via RT-qPCR. **B**, **C** The viability and proliferation of transfected cells were determined with CCK-8 and colony formation assays. **D** Flow cytometry analysis showed the apoptosis in each group. **E**, **F** Migration and invasion of cells with indicated transfections were measured by wound healing assay and Transwell assays. **G** EMT-associated proteins in the HCC1937 cell lines of indicated groups were tested by western blot. *p < 0.05, **p < 0.01, ***p < 0.001

Finally, rescue assays were conducted to further verify that mechanisms of PITPNA-AS1 mentioned above could drive the development of TNBC. Accordingly, SIK2 expression was upregulated in HCC1937 cells by transfecting pcDNA3.1/SIK2 (Fig. 7A). Through results of CCK-8 and colony formation assays, we found that cell viability and proliferation were inhibited by PITPNA-AS1 deficiency and was promoted by transfection of pcDNA3.1-SIK2 or cotransfection of miR-520d-5p inhibitor + pcDNA3.1-DDX54. The suppressive effects of PITPNA-AS1 deficiency onHCC1937 cell viability and proliferation were rescued by SIK2 overexpression or by upregulated DDX54 after inhibiting miR-520d-5p (Fig. 7B, C). Transfection of pcDNA3.1-SIK2 or cotransfection of miR-520d-5p inhibitor + pcDNA3.1-DDX54 inhibited apoptosis rate of HCC1937 cells. Cell apoptosis induced by silenced PITPNA-AS1 was inhibited by upregulated SIK2; this effect was also reversed by co-effect of suppressed miR-520d-5p and overexpressed DDX54 (Fig. 7D). In addition, cell migration and invasion inhibited by PITPNA-AS1 knockdown were offset by overexpressing SIK2 or by inhibiting miR-520d-5p expression together with upregulating DDX54 (Fig. 7E, F). Moreover, either SIK2 overexpression or miR-520d-5p inhibition together with DDX54 upregulation rescued the effect of silenced PITPNA-AS1 on the EMT process (Fig. 7G).

### MYBL2 transcriptionally activated PITPNA-AS1 expression in TNBC

Transcription activation is a major cause for the aberrant expression of genes. Considering this, we explored whether upregulation of PITPNA-AS1 is attributed to this manner. Through UCSC (http://genome.ucsc.edu/), some potential transcription factors that could bind to PITPNA-AS1 promoter were found. Among which, E2F6, PAX5, FOXP2, ELK5, MYBL2, YY1 were found at a higher level in TNBC cells than in MCF10A cell line, as revealed by RT-qPCR analysis (Additional file 1: Figure S1A). Later, above-mentioned transcription factors were upregulated in HCC1937 and MDA-MB-468 cells by transfection of pcDNA3.1 overexpression plasmid for further analysis (Additional file 1: Figure S1B). Observed from Additional file 1: Figure S1C, we found that PITPNA-AS1 expression was predominantly increased in MYBL2-overexpressed cells. Further, we knocked down MYBL2, and discovered that MYBL2 silencing caused downregulation in PITPNA-AS1 expression level (Additional file 1: Figure S1D). Therefore, we hypothesized that PITPNA-AS1 could be transcriptionally activated by MYBL2 in TNBC cells. MYBL2 DNA motif and four putative binding sites of MYBL2 on PITPNA-AS1 promoter were predicted based on Jaspar online database (Additional file 1: Figure S1E). ChIP assay suggested the binding of MYBL2 to PITPNA-AS1 promoter in P1 section which contained site 1 and site 2 (Additional file 1: Figure S1F). Next, luciferase reporter assay was performed to further confirm the interaction of MYBL2 and PITPNA-AS1 promoter. We discovered that the luciferase activity of vectors containing WT and Mut 1 sequences of PITPNA-AS1 promoter was strengthened while that of vectors containing Mut 2 and Mut 1/2 sequences of PITPNA-AS1 promoter remained unchanged after overexpressing MYBL2, indicating that MYBL2 interacted with PITPNA-AS1 promoter at site 2 (Additional file 1: Figure S1G).

## Discussion

Increasing literatures reported that lncRNAs are involved in occurrence and development of various human cancers by sponging miRNA or recruiting RBPs [23, 24]. Although the essential roles of abnormally expressed lncRNAs have been highlighted in TNBC progression [14, 25], the potential role and mechanism of lncRNA PITPNA-AS1 underlying TNBC remain obscure and deserve to be explored. Our study suggested that PITPNA-AS1 expression was higher in TNBC tissues and cells than in control tissues and cells, and mainly distributed in the cytoplasm of TNBC cells. Moreover, decreased PITPNA-AS1 repressed TNBC cell proliferation, facilitated cell apoptosis, and suppressed cell migration and invasion in vitro. Furthermore, PITPNA-AS1 silencing inhibited xenograft tumor growth in vivo. Collectively, PITPNA-AS1 exhibited oncogenic properties in TNBC.

MiRNAs are another class of ncRNAs with about 22–24 nucleotides in length and played important roles in cancer progression [26, 27]. Existing evidence has depicted that lncRNAs can combine with specific miRNA to facilitate or suppress the initiation or progression of tumors [28, 29]. In this study, miR-520d-5p was identified for further exploration via bioinformatics analysis and a series of molecular mechanism experiments. Previously, miR-520d-5p was found to inhibit cell proliferation and cell cycle via targeting PTTG1 in glioma [30]. In gastric cancer, miR-520d-5p is an important regulator in cell proliferation and survival [31]. In addition, miR-520d-5p functions as an anti-oncogene in colorectal cancer and suppresses tumor growth and metastasis via regulating CTHRC1 [32]. Herein, we found that miR-520d-5p had binding capacity with PITPNA-AS1 in TNBC. Furthermore, miR-520d-5p expression was at a low level in TNBC tissues and cell lines. These findings suggested that PITPNA-AS1 sequestered miR-520d-5p in TNBC.

Salt inducible kinase 2 (SIK2) has been validated to exert tumor-promoting functions in a variety of cancers, including TNBC [33, 34]. Nevertheless, the relationship between SIK2 and miR-520d-5p (or PITPNA-AS1) in TNBC cells needs investigation. In our study, it was verified that SIK2 was directly targeted by miR-520d-5p in TNBC. More importantly, results in our study indicated that PITPNA-AS1 positively modulated SIK2 expression not merely via sponging miR-520d-5p.

DEAD-box helicase 54 (DDX54) was recognized as a member of RBPs and reported as an oncogene in some cancers [35, 36]. In this study, SIK2 mRNA stability was inhibited by PITPNA-AS1 knockdown. DDX54 was identified to interact with PITPNA-AS1 (or SIK2) in TNBC cells. In addition, restoration experiments suggested that TNBC cellular processes inhibited by silenced PITPNA-AS1 was rescued by SIK2 overexpression or co-effect of miR-520d-5p inhibition and DDX54 upregulation.

Emerging investigations have implied that transcriptional regulation mediated by transcription factor was a major reason for the aberrant expression of lncRNAs [37, 38]. MYB proto-oncogene Like 2 (MYBL2) was known as a transcription factor in lung adenocarcinoma [39]. Our study revealed that MYBL2 positively regulated PITPNA-AS1 expression and bound to PITPNA-AS1 promoter, which indicated that the upregulation of PITPNA-AS1 in TNBC was transcriptionally induced by MYBL2.

## Conclusions

Conclusively, our study showed that MYBL2-induced PITPNA-AS1 upregulated SIK2 expression to drive TNBC cellular processes via miR-520d-5p and DDX54. This might provide a meaningful theoretic basis for further exploration on TNBC therapy strategies.

## Supplementary Information

**Additional file 1: Figure S1.** The upregulation of PITPNA-AS1 was transcriptionally induced by MYBL2. (A) Six putative transcription factors for PITPNA-AS1 with high level in TNBC cells. (B) The overexpression efficiency of mentioned-above transcription factors in HCC1937 and MDA-MB-468 cells. (C) PITPNA-AS1 expression after overexpression of the 6 predicted transcription factors was detected by RT-qPCR. (D) MYBL2 and PITPNA-AS1 expression levels in TNBC cells transfected with sh-MYBL2 or sh-NC were measured through RT-qPCR. (E) MYBL2 DNA motif and binding sites of MYBL2 to PITPNA-AS1 promoter. (F) ChIP assay was conducted to determine the binding between MYBL2 and PITPNA-AS1 promoter. (G) The binding site between MYBL2 and PITPNA-AS1 promoter was confirmed by luciferase reporter assay. **p < 0.01, ***p < 0.001.

## Data Availability

All data from this study are available in this published article.

## Abbreviations

lncRNAs: Long non-coding RNAs
PITPNA-AS1: LncRNA PITPNA antisense RNA 1
BC: Breast cancer
ER: Estrogen receptor
PR: Progesterone receptor
TNBC: Triple-negative breast cancer
ncRNAs: Non-coding RNAs
ceRNA: Competing endogenous RNA
miRNA: MicroRNA
RBPs: RNA-binding proteins
