# Supplementary figures and images for: MYBL2-induced PITPNA-AS1 upregulates SIK2 to exert oncogenic function in triple-negative breast cancer through miR-520d-5p and DDX54
Source: J Transl Med. 2021 Aug 5;19:333. doi: 10.1186/s12967-021-02956-6 (PMC8340450; doi:10.1186/s12967-021-02956-6)

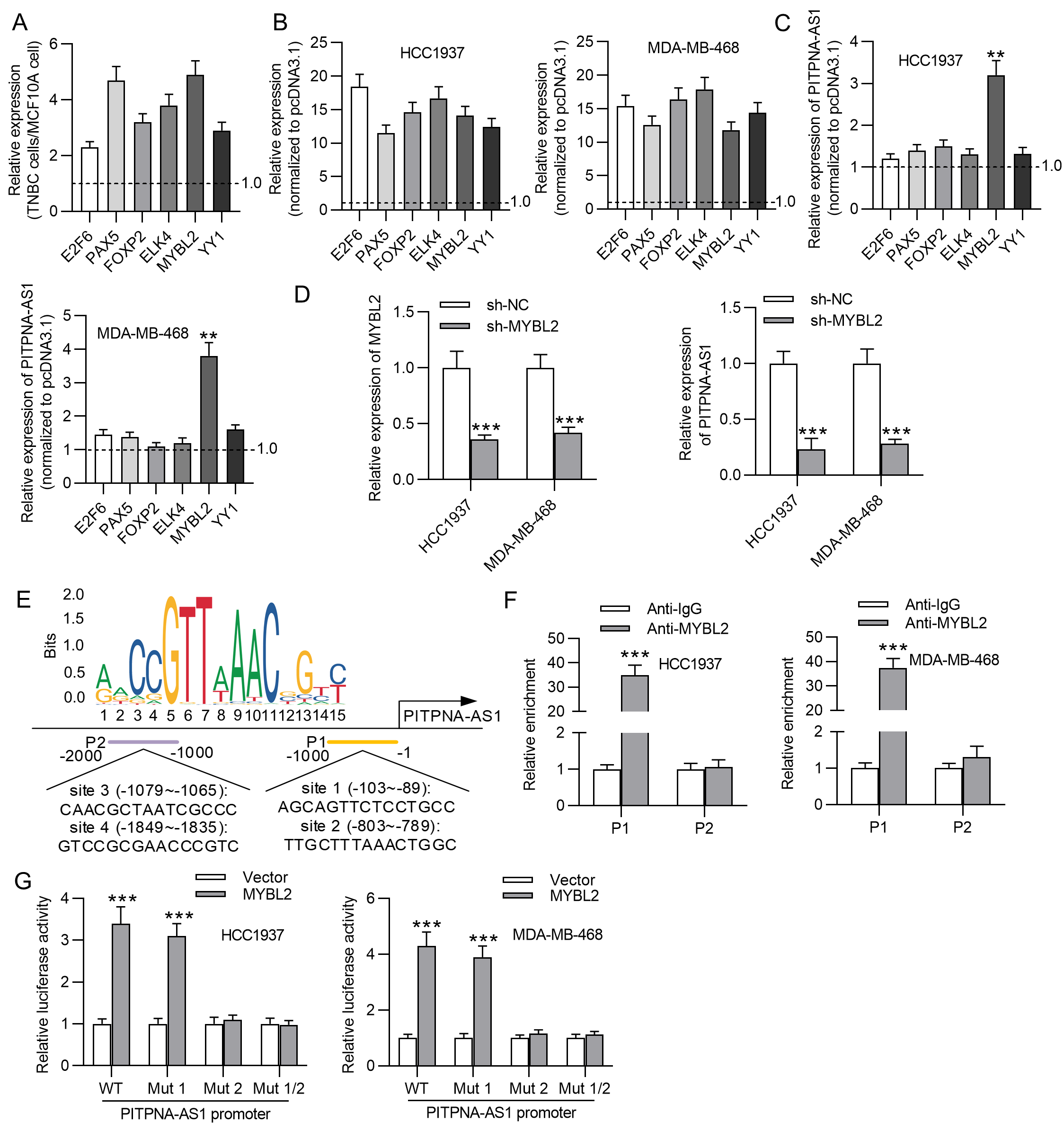

Supplement: Supplementary file 1 — Additional file 1: Figure S1. The upregulation of PITPNA-AS1 was transcriptionally induced by MYBL2. (A) Six putative transcription factors for PITPNA-AS1 with high level in TNBC cells. (B) The overexpression efficiency of mentioned-above transcription factors in HCC1937 and MDA-MB-468 cells. (C) PITPNA-AS1 expression after overexpression of the 6 predicted transcription factors was detected by RT-qPCR. (D) MYBL2 and PITPNA-AS1 expression levels in TNBC cells transfected with sh-MYBL2 or sh-NC were measured through RT-qPCR. (E) MYBL2 DNA motif and binding sites of MYBL2 to PITPNA-AS1 promoter. (F) ChIP assay was conducted to determine the binding between MYBL2 and PITPNA-AS1 promoter. (G) The binding site between MYBL2 and PITPNA-AS1 promoter was confirmed by luciferase reporter assay. **p < 0.01, ***p < 0.001. [file 12967_2021_2956_MOESM1_ESM.tif]
